# Combined Systematic and MRI-US Fusion Prostate Biopsy Has the Highest Grading Accuracy When Compared to Final Pathology

**DOI:** 10.3390/medicina57060519

**Published:** 2021-05-22

**Authors:** Iulia Andras, Emanuel Darius Cata, Andreea Serban, Pierre Kadula, Teodora Telecan, Maximilian Buzoianu, Maria Bungardean, Dan Vasile Stanca, Ioan Coman, Nicolae Crisan

**Affiliations:** 1 Department of Urology, Faculty of Medicine, Iuliu Hatieganu University of Medicine and Pharmacy, 400012 Cluj-Napoca, Romania; dr.iuliaandras@gmail.com (I.A.); serban_andreea95@yahoo.com (A.S.); maximilian.buzoianu@yahoo.com (M.B.); vasilestanca@yahoo.com (D.V.S.); jcoman@yahoo.com (I.C.); drnicolaecrisan@gmail.com (N.C.); 2 Department of Urology, Municipal Hospital, 400139 Cluj-Napoca, Romania; pakadula@gmail.com; 3 Department of Pathology, Faculty of Medicine, Iuliu Hatieganu University of Medicine and Pharmacy, 400012 Cluj-Napoca, Romania; mariabun2002@yahoo.com; 4 Department of Pathology, Emergency Country Hospital, 400006 Cluj-Napoca, Romania

**Keywords:** Gleason group, MRI, MRI-US fusion prostate biopsy, radical prostatectomy

## Abstract

*Background and objectives:* Systematic prostate biopsy (SB) has a low Gleason group (GG) accuracy when compared to final pathology. This may negatively impact the inclusion of patients into specific risk groups and treatment choice. The aim of our study was to assess the GG accuracy of magnetic resonance imaging-ultrasound (MRI-US) fusion prostate biopsy. *Materials and Methods:* Of a cohort of minimally invasive radical prostatectomy (RP), we selected all patients who were diagnosed with prostate cancer (PCa) via MRI-US fusion biopsy (*n* = 115). *Results:* Combined biopsy had the highest rate for GG concordance (61.7% vs. 60.4% for SB vs. 45.3% for MRI-US fusion biopsy) and the lowest for upgrading (20.9% vs. 24.5% for SB vs. 34.9% for MRI-US fusion biopsy), *p* < 0.0001. No clinical data were predictive for upgrading or downgrading at final pathology. Locally advanced PCa was associated with a high Prostate Imaging-Reporting and Data System (PIRADS) score (*p* = 0.0014) and higher percentages of positive biopsy cores (PBC)/targeted (*p* = 0.0002) and PBC/total (*p* = 0.01). Positive surgical margins were correlated with higher percentages of PBC/systematic (*p* = 0.003) and PBC/total (*p* = 0.009). *Conclusions:* Pre-biopsy prostate MRI improves GG concordance between biopsy and RP. Combined biopsy provides the highest grading accuracy when compared to final pathology. Targeted and systematic biopsy data are predictive for adverse pathologic outcomes.

## 1. Introduction

Until recently, systematic ultrasound-guided prostate biopsy was considered the gold standard for prostate cancer (PCa) diagnosis. Although it entails the sampling of the whole prostate, it is hampered by a low sensitivity and specificity for the detection of PCa [1]. Up to 50% of patients with one negative systematic biopsy will be diagnosed with PCa during follow-up [2]. In other words, an important percentage of the patients with PCa have a false negative result, leading to the underdiagnosis of a potentially lethal disease. Furthermore, by unintentionally missing the index lesion due to the diagnostic limitations of the ultrasound, the systematic biopsy may portray a false image of the disease, leading to inaccurate inclusion into the correct risk group and under- or overtreatment. A recent retrospective analysis of a large cohort (17,598 patients) showed a 25.5% rate of upgrading and 15.6% rate of downgrading when comparing systematic biopsy to radical prostatectomy (RP) specimen [3]. Therefore, a more accurate characterization of PCa is needed for the correct treatment choice.

Magnetic resonance imaging (MRI)-guided prostate biopsy has recently been introduced in the diagnostic pathway of PCa [4]. All three types of targeted biopsies (cognitive, MRI-ultrasound (US) fusion and in-bore) have shown increased detection rates for clinically significant disease compared to systematic biopsy [5]. By targeting the index lesion, which is considered the main determinant of PCa behavior [6], MRI-guided biopsy should lead to a more accurate prediction of the final pathologic grading and oncologic outcomes after treatment. Gandaglia et al. have shown that the combination of MRI and MRI-targeted biopsy with systematic biopsy data into a predictive model has higher accuracy for the prediction of biochemical recurrence following RP (77%) when compared to current risk stratification (62%) [7].

The aim of our study was to assess the accuracy of Gleason group (GG) grading of MRI-US fusion guided prostate biopsy compared to RP specimen. A secondary objective was to identify any predictors of unfavorable pathologic outcomes.

## 2. Materials and Methods

### 2.1. Study Population

**Figure 1:**
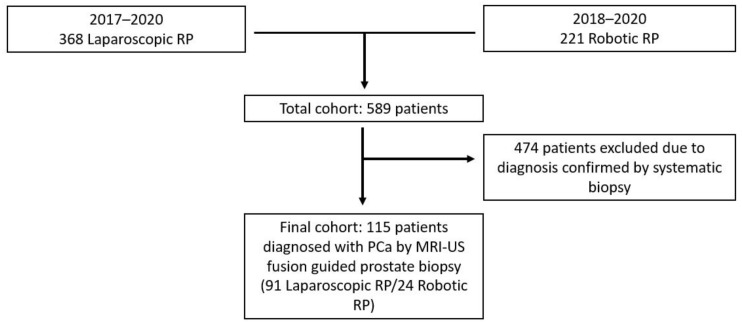
Flow diagram illustrating cohort selection for the current study.

Of a consecutive series of minimally invasive radical prostatectomy (RP), we selected all patients who were diagnosed with PCa via MRI-US fusion prostate biopsy (*n* = 115, Figure 1). The study was conducted in accordance with the Declaration of Helsinki and was approved by the Institutional Ethics Committee.

### 2.2. MRI and MRI-US Fusion Prostate Biopsy Protocol

About a third of MRIs (29.6%, *n* = 34) were performed in our center and interpreted by 3 radiologists with 5 years of experience, whereas 70.4% (*n* = 81) were performed in external centers (no data are available regarding the experience of the radiologists). The indication for prostate MRI was any suspicion for PCa: prostate-specific antigen (PSA) ≥ 4 ng/mL and/or positive digital rectal examination. All imaging was performed on 1.5 T field-strength MRI and respected Prostate Imaging-Reporting and Data System (PIRADS) v2 or v2.1 recommendation. Endorectal coil was used at radiologists’ discretion. The presence of a lesion with a PIRADS score ≥ 3 was considered the threshold for biopsy in all patients.

MRI-US fusion prostate biopsy was performed by two urologists in the same center, using a transrectal approach. During the study period, no other centers in our area performed any type of MRI-guided prostate biopsy. Since 2017, all patients in our department have undergone MRI-US fusion prostate biopsy in case of suspicious MRI findings. The biopsies performed during the learning curve of the two urologists were included in the current analysis. The Hitachi Arietta 70a system with real-time virtual sonography (RVS) software and rigid registration was employed. Local anesthesia was performed in all patients by endorectal instillation of lidocaine gel. The MRI-US fusion biopsy was either primary (73% of cases) or in repeat setting after a previous negative systematic biopsy (27% of cases). The biopsy began with 1–4 targeted cores, followed by systematic 12-core sampling. The same urologist performed both targeted and systematic biopsies and was aware of the patient’s clinical and imaging data.

### 2.3. Surgical Approach

RP was performed either through a 3D properitoneal laparoscopic or robotic transperitoneal approach. The choice of approach was at patients’ discretion, as the robotic approach was available only in a private hospital.

### 2.4. Pathology

Pathology assessment of biopsy and RP specimens was performed by the same three pathologists (one senior and two young pathologists supervised by the senior).

### 2.5. Cohort and Definitions

PCa with GG 3 or higher was defined as high-grade (HG) disease, and GG of 2 or less was defined as low-grade (LG) disease. This definition was chosen due to the significantly worse oncologic outcomes of patients with GG3 disease and higher, compared to GG2 [8,9]. Downgrading was defined as the presence of an inferior GG, and upgrading was defined as the presence of a superior GG on the RP specimen compared to the biopsy. A GG concordance was considered when both RP and biopsy GG were identical. Positive surgical margin at RP was defined as the presence of tumor tissue at the inked border of the specimen. Favorable PCa was defined as pT2 and GG 1 or 2.

### 2.6. Statistical Analysis

Statistical analysis was performed using MedCalc software (Oostend, Belgium). Continuous variables were presented as median and interquartile range, whereas categorial variables as frequencies and proportions. A Chi-square test was used to assess the differences between categorical variables. Analysis of variance of continuous variables was performed using a Kruskal–Wallis test according to data distribution. *p* < 0.05 was considered statistically significant.

## 3. Results

### 3.1. General Characteristics

The study cohort included 115 patients. The general characteristics are summarized in Table 1. The majority of the patients had LG disease on combined (75.9%), targeted (75.6%), and systematic biopsy (76.4%). MRI-US fusion biopsy was negative for PCa in 29 cases, whereas systematic biopsy was negative in 9 cases.

The median time between biopsy and RP was 3 months, interquartile range (IQR) 2–4. Pathology confirmed the presence of LG disease in 79.1% of the cases, whereas 20.9% harbored HG PCa. Favorable disease was present in 66.1% of the patients. Twenty-nine patients (25.21%) had extracapsular PCa. The overall rate of positive surgical margins (PSM) was 20.86%.

The patients with negative MRI-US fusion biopsy had LG PCa on RP specimen in 79.31% of cases, whereas the other 20.68% harbored HG disease. Organ-confined PCa was identified in 93.1% and favorable disease in 75.86% of these patients. The patients with negative systematic biopsy harbored LG in 88.89% and HG disease in 11.11% of cases. pT2 and favorable disease were confirmed in 83.33% of the patients.

### 3.2. Biopsy GG vs. Radical Prostatectomy GG

The overall rate of GG concordance between prostate biopsy and RP specimen was 61.7%, the upgrading rate was 20.9%, and the downgrading rate was 17.4%.

We observed a lower GG concordance rate between MRI-US fusion cores and RP (45.3%) compared to systematic biopsy (60.4%), *p* < 0.0001. However, the addition of MRI-targeted biopsy cores to the systematic sampling led to an increase in the concordance and a decrease in the upgrading rate when compared to RP specimen (Table 2). Combined MRI-US fusion and systematic biopsy led to the highest rates of concordance and the lowest of upgrading between biopsy and RP. The lowest rates for downgrading were observed for systematic sampling.

### 3.3. Predictors of Upgrading/Downgrading

We observed no significant differences between patients who were concordant, upgraded, or downgraded in terms of PSA, PSA density, previous biopsy, lesion location, dimension of lesion, PIRADS score, number of targeted biopsy cores, maximum cancer core length (MCCL), and percentage of positive biopsy cores (PBC) out of total/targeted/systematic cores (Table 3). Prostate volume was significantly higher in patients who were upgraded (median 52 g) or downgraded (median 42.17 g) compared to the ones that had similar GG on biopsy and RP specimen (median 39.92 g), *p* = 0.03.

In patients with HG on biopsy, we observed lower rates of any downgrading on RP specimen if HG was present on both systematic and targeted cores (50%), compared to HG present on systematic (56%) or MRI-US fusion cores (52.38%), but the data did not reach statistical significance (*p* = 0.712).

### 3.4. Predictors of Unfavorable RP Pathologic Outcomes

Patients with higher PIRADS score harbored a higher rate of locally advanced PCa (15.38%, 5.71% and 41.93%, for PIRADS 3, 4 and 5, respectively, *p* = 0.0014) and unfavorable disease (15.38%, 22.85% and 45.16% for PIRADS 3, 4 and 5, respectively, *p* = 0.06). Also, higher rates of PSM were observed with rising PIRADS score, although not statistically significant (7.69%, 20% and 29.03% for PIRADS 3, 4 and 5, respectively, *p* = 0.27).

The presence of concurrent HG on targeted and systematic cores was correlated with a higher rate of locally advanced PCa (68.75% vs. 52% for HG on systematic vs. 57.14% for HG on targeted cores, *p* = 0.07) and unfavorable disease (81.25% vs. 76% for HG on systematic biopsy vs. 66.66% for HG on targeted cores, *p* = 0.04). However, the rate of PSM was not significantly different in patients with HG disease on MRI-US fusion cores, systematic cores, or on both targeted and systematic biopsies (23.8% vs. 36% vs. 25%, *p* = 0.23).

Patients with pT3 vs. pT2 PCa had a higher percentage of PBC/systematic (41.67% vs. 25%, *p* = 0.23), higher percentage of PBC/targeted cores (100% vs. 50%, *p* = 0.0002), and higher percentage of PBC/total (40% vs. 28.57%, *p* = 0.01). Unfavorable disease was associated with higher percentage of PBC/systematic/targeted/total biopsy cores (33.33%/75%/40% vs. 25%/50%/28.57% for favorable disease; *p* = 0.3, *p* = 0.018 and *p* = 0.05, respectively). Similarly, the percentage of PBC/systematic/targeted/total was higher for patients with PSM (41.67%/83.35%/43.75% for PSM vs. 25%/50%/31.25% for patients with negative surgical margins, *p* = 0.003, *p* = 0.413 and *p* = 0.009, respectively).

MCCL on systematic/targeted/combined biopsy was significantly associated with the presence of locally advanced disease: 8 (IQR 5–12)/7(IQR 5.2–11)/9 (IQR 6–13.2) for pT3 vs. 5 (IQR 3–9)/5 (IQR 1.2–8)/5 (IQR 4–9) for pT2, *p* = 0.01/0.01/0.003, respectively. Unfavorable disease had higher MCCL on targeted biopsy than favorable disease (7 (IQR 5–11) vs. 5 (IQR 2–8), respectively, *p* = 0.04). MCCL on systematic or combined biopsy was not correlated with unfavorable disease (*p* = 0.22 and *p* = 0.07). Additionally, PSM were not associated with either MCCL on systematic/targeted/combined biopsy, *p* = 0.33/0.6/0.188, respectively.

## 4. Discussion

Our study shows that the addition of MRI-targeted biopsy cores to systematic biopsy improves the concordance rate between prostate biopsy and RP specimen. Lower rates of upgrading were seen for combined biopsy, whereas a higher downgrading rate was observed for MRI-US fusion biopsy when compared with a systematic or combined approach. One headline of our study is represented by the apparent good accuracy of systematic biopsy. However, we chose not to emphasize this outcome, as the systematic sampling performed in our cohort is not reflective of the traditional systematic biopsy. Both targeted and systematic cores were taken by the same operator, who was aware of the MRI result. Additionally, the systematic sampling was semicognitive, aiming to overcome errors of the technique (rigid registration) and errors of the operator, as we included cases performed during the learning curve in the current cohort biopsy.

Several retrospective cohorts have shown similar results for combined biopsy when comparing to RP specimen [10,11]. Diamand et al. concluded that despite performing only three additional cores via targeted biopsy, the combination between systematic and MRI-guided sampling increased concordance with final histology from 49.4% to 63.2% for overall PCa and from 41.2% to 56.7% for clinically significant PCa in a group of 443 patients [10]. Moreover, the combination of MRI-US fusion biopsy and saturation biopsy of the whole prostate (24 cores) led to improvement in the accuracy of index lesion detection when confronted with RP [12]. Radtke et al. showed that MRI-US fusion biopsy diagnosed 80% of index lesions, saturation biopsy detected 92%, whereas their combination reached a diagnosis rate of 96% [12].

Opposite results have been published on whether systematic or targeted biopsy has the highest GG concordance rate with final pathology [13,14,15]. Knowledge of MRI results prior to the procedure can lead to a good GG accuracy of systematic biopsy due to cognitive guidance of systematic cores close to the index lesion, as was the case in our study. When systematic biopsy is performed blinded to the MRI result or without prior imaging, it shows lower GG concordance with RP compared to targeted biopsy [13,16,17,18]. Interestingly, Borkowetz et al. showed that, despite availability of MRI data to the urologist performing the systematic biopsy, targeted biopsy was more accurate than systematic sampling, showing higher GG concordance and lower upgrading rates to RP [19]. However, it is of note that in this cohort, 51% of the index lesions were located in the anterior transitional zone, out of the standard systematic reach [19].

Reduction in the upgrading rate from biopsy to final pathology can be accomplished by a more accurate targeting of the index lesion in order to minimize the impact of tumor heterogeneity. One possibility is to perform saturation biopsy of the index lesion: biopsy at every 6 mm along the axis of maximum diameter [20]. Using this approach, Calio et al. showed a decrease in the upgrading rate from 18% in patients without saturation biopsy to 7% when saturation biopsy of the index lesion was performed (*p* = 0.021) [20]. Furthermore, Ploussard et al. showed that obtaining a minimum of 4 cores in the case of 3 PIRADS lesions and a minimum of 3 cores in the case of 4–5 PIRADS lesions can lead to a 30% reduction in the upgrading rate [21]. However, in current clinical practice, the urologist performing the systematic biopsy is not blinded to the MRI, and frequently, the systematic targeting becomes cognitive-guided. As such, it may be considered almost a surrogate for saturation biopsy of the index lesion and a method to overcome errors of image registration or targeting [22].

We observed the highest downgrading rate for targeted biopsy when compared to systematic or combined sampling. In a large retrospective analysis of 10,220 patients, pre-biopsy MRI was associated with significantly increased odds for downgrading [23], whereas Kayano et al. reported systematic biopsy as the only predictive factor for upgrading both in univariate and multivariate analysis [13]. Increased rates of downgrading were reported in the case of both in-bore targeted biopsy and fusion targeted biopsy, which are caused by oversampling of aggressive areas [10,19,24,25]. A higher GG on prostate biopsy leads to overtreatment, with the associated negative impact of long-term sequela after radical treatment [26]. As such, the exact number of targeted cores that should be obtained from a lesion is yet to be determined. When HG PCa is identified both on systematic and targeted biopsy, there is a significantly lower chance of downgrading and a significantly higher risk of aggressive pathological characteristics (pT3-T4, pN1, positive surgical margins) [27].

We identified no predictive factors for the upgrading or downgrading rate, similar to Le et al., which may be due to the low number of patients in both cohorts [11]. Increased prostate volume led to a lower grading accuracy in our cohort. In larger prostates, there is a higher chance of missing the index lesion (thus leading to a high upgrading rate) or missing other cancer sites (which might downgrade the final pathology result). Similar results have been reported by other authors. Stackhouse et al. [28] reported prostate weight as an independent prediction factor for undergrading in the case of systematic biopsy, whereas Epstein et al. [29] showed that higher prostate weight was associated with higher upgrading rate in a cohort of 7643 patients. However, in more recent studies analyzing targeted biopsy [10,30], prostate volume was not a predictive factor for upgrading or downgrading rate in either univariate or multivariate analysis. On the other hand, previous reports identified history of negative biopsy and number of cores as significant predictors for GG concordance between biopsy and RP [10].

An important observation of our study is that when both biopsy approaches identify HG disease, there is a higher likelihood that locally advanced PCa and unfavorable disease are confirmed. Manceau et al. showed that concurrent presence of HG on targeted and systematic biopsy was also correlated with PSM, which was not confirmed in our study [27]. The inclusion of highest targeted biopsy GG, along with preoperative PSA and perineural invasion on targeted cores, in a nomogram has shown a high accuracy (72.4–76.6%) for the prediction of advanced PCa (pT3-T4 +/− pN1) [31].

Furthermore, a higher percentage of PBC/targeted and a higher MCCL on systematic/targeted/combined biopsy cores suggest that pT3 disease is more likely to be present, while a higher MCCL on targeted cores is associated with unfavorable disease. Furthermore, a higher percentage of PBC/systematic is associated with higher rate of PSM, suggesting a high-volume disease. Patel et al. reported that 70% of the PSM were located in the vicinity of the index lesion identified by mpMRI [32]. However, utilization of mpMRI does not reduce the risk of PSM, as shown in a randomized clinical trial [33], probably due to underestimation of pathological tumor size [34]. Tumor volume has previously been shown to be associated with GG, number of PBC, percentage of tumor length, tumor bilaterality, and MCCL on targeted biopsy [35,36,37]. Higher percentage of PBC/total marks a high-volume PCa and was correlated with locally advanced stage and PSM in our cohort. Higher rates of PSM were also reported in patients with higher tumor volume [38] or in those with lower total prostate volume [39].

Our study is not devoid of limitations. First of all, it is a retrospective study on a relatively small sample size. Furthermore, we included a mixed cohort of patients with single or multiple biopsies, which may have led to a low rate of clinically significant PCa. Moreover, the same urologist performed both the systematic and targeted biopsies, tailoring the systematic sampling in dependance to the mpMRI information, leading to superior outcomes as compared to targeted cores. Finally, we included biopsies performed during the learning curve of the two urologists, so systematic sampling had the purpose of accounting for any targeting error.

In conclusion, we consider that pre-biopsy MRI increases the concordance between prostate biopsy and RP specimen. The association between MRI-US fusion prostate biopsy and systematic sampling provides the highest grading accuracy when compared to final pathology. The PIRADS score, the presence of concurrent high-grade disease on targeted and systematic cores, and percentage of positive biopsy cores out of targeted/systematic/total cores are correlated with adverse pathologic outcomes.

## Figures and Tables

**Table 1:** General characteristics of the cohort.

Variables		Value (IQR)	
Age, years Median (IQR)		64 (60–67)	
PSA, ng/mL Median (IQR)		8 (5–10.1)	
Prostate volume, g Median (IQR)		41 (33–52)	
PSA density, ng/mL/g Median (IQR)		0.17 (0.12–0.28)	
Lesion dimension on MRI, mm Median (IQR)		15 (10–21)	
PIRADS score *		3—16.5% 4—44.3% 5—39.2%	
Number of targeted biopsy cores Median (IQR)		3 (3–4)	
Biopsy GG	Combined biopsy GG 1—25.5% 2—50.4% 3—14.8% 4—4.3% 5—5.2%	MRI-US fusion GG 1—33.72% 2—41.86% 3—10.46% 4—9.3% 5—4.65%	Systematic biopsy GG 1—28.3% 2—48.11% 3—12.26% 4—6.89% 5—3.77%
Maximum cancer core length, mm Median (IQR)	Combined biopsy 6 (4–11)	MRI-US fusion biopsy 6 (3–9)	Systematic biopsy 5 (3–9)
Radical prostatectomy GG		1—16.5% 2—62.6% 3—15.7% 4—0 5—5.2%	
pT		pT2—86 (74.78%) pT3—29 (25.21%)	
Surgical margins	Overall R0—79.14% R1—20.86%	pT2 R0—83.73% R1—16.27%	pT3 R0—65.52% R1—34.48%

* PIRADS score is reported according to v2 or v2.1, depending on the available guidelines during the period when the MRI was performed. IQR = interquartile range.

**Table 2:** Concordance rates between GG of MRI-US fusion prostate biopsy and radical prostatectomy specimen.

	MRI-US Fusion Biopsy	Systematic Biopsy	Combined Biopsy	*p*
Concordance, *n* (%)	39 (45.3%)	64 (60.4%)	71 (61.7%)	<0.0001
Upgrading, *n* (%)	30 (34.9%)	26 (24.5%)	24 (20.9%)	
Downgrading, *n* (%)	17 (19.8%)	16 (15.1%)	20 (17.4%)	

**Table 3:** Comparison between the characteristics of patients who were upgraded, downgraded, and concordant in terms of GG with final pathology.

Variable	Upgraded	Downgraded	Concordant	*p*
PSA, ng/mL Median (IQR)	9 (5.7–12.8)	8.8 (5.3–10.2)	7.5 (4.8–10)	0.39
Prostate volume, g Median (IQR)	52 (37.5–50.2)	42.1 (37.5–50.2)	39.9 (28.8–46.7)	0.03
PSA density, ng/mL/g Median (IQR)	0.17 (0.14–0.23)	0.2 (0.09–0.26)	0.17 (0.12–0.32)	0.9
Previous negative biopsy	Yes 75% No 25%	Yes 65% No 35%	Yes 74.6% No 25.4%	0.67
Lesion location on MRI	Anterior 13.04% Transitional 30.43% Peripheral 56.52%	Anterior 5.26% Transitional 26.31% Peripheral 68.42%	Anterior 7.35% Transitional 25% Peripheral 67.64%	0.83
Lesion dimension on MRI, mm Median (IQR)	19 (12.1–24.2)	12 (9–20.2)	15 (12–19)	0.28
PIRADS score *	3—9.52% 4—38.09% 5—52.38%	3—16.66% 4—58.33% 5—25%	3—19.56% 4—43.47% 5—36.95%	0.52
Number of targeted biopsy cores Median (IQR)	3 (3–4)	3 (3–3)	3 (3–4)	0.89
%PBC/total Median (IQR)	29.91 (13.3–43.3)	27.62 (16.6–40)	33.33 (21.4–53.3)	0.24
%PBC/targeted Median (IQR)	41.66 (0–100)	66.67 (0–100)	50 (25–100)	0.8
%PBC/systematic Median (IQR)	20.83 (8.3–41.6)	25 (16.6–33.3)	33.33 (16.6–50)	0.21
MCCL on combined biopsy, mm Median (IQR)	5 (2.5–11.5)	6.5 (4.5–8)	6 (4–11)	0.48
MCCL on targeted biopsy, mm Median (IQR)	5 (2.6–9.5)	6 (5–7)	6 (3–9)	0.96
MCCL on systematic biopsy, mm Median (IQR)	5 (3–9)	4 (2.2–6.5)	6 (4–11)	0.13

* PIRADS score is reported according to v2 or v2.1, depending on the available guidelines during the period when the MRI was performed. IQR = interquartile range.

## Data Availability

All the data supporting the result are available upon request from the corresponding author.
